# Molecular aspects of sGC regulation

**DOI:** 10.1186/1471-2210-11-S1-O10

**Published:** 2011-08-01

**Authors:** Michael A Marletta, Eric S Underbakke, Nathaniel B Fernhoff

**Affiliations:** 1 University of California, Berkeley, Berkeley, CA 94720-3220, USA

## 

**Figure 1:**
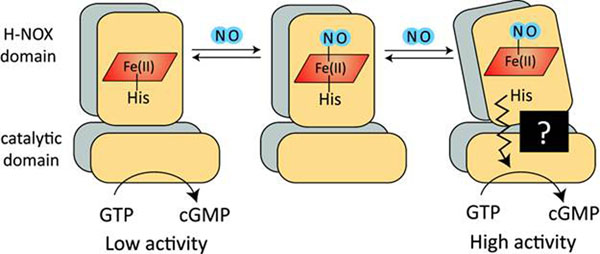
Schematic of sGC regulation by NO. In the resting, unliganded state, sGC has a low basal activity. The addition of NO leads initially to a 6-coordinate complex that then forms a highly active 5-coordinate complex. Evidence supports an additional requirement for NO that accelerates the formation of the 5-coordinate complex and fully activates the enzyme.

Mammalian sGC is a heterodimer composed of α- and β-subunits (Figure 1) [1]. The C-terminus of each subunit contains a catalytic domain, and the active site is composed of residues from both subunits. Sequence analysis shows that each subunit also contains a well-defined PAS-like domain, and a predicted helical region. The N-termini of the α- and β-subunits are homologous to the H-NOX (Heme-Nitric oxide/OXygen) family of proteins. The N-terminus of β-subunit contains a ferrous heme cofactor that serves a receptor for NO.

Ferric heme oxidized sGC has low activity, and the NO complex of the re-reduced heme generates a desensitized, low-activity state of sGC. The molecular mechanism for this desensitization involves site specific S-nitrosation. The conformational changes associated with activation are both subtle and complex. Hydrogen-deuterium exchange mass spectrometry analysis can be used to probe conformational changes and protein-protein interactions. This method has been brought to bear on sGC, illuminating domain interactions within sGC and conformational changes induced by NO binding.
